# Improving Patient Access and Reducing Costs for Glaucoma with Integrated Hospital and Community Care: A Case Study from Australia

**DOI:** 10.5334/ijic.4642

**Published:** 2019-11-06

**Authors:** Belinda K. Ford, Blake Angell, Gerald Liew, Andrew J. R. White, Lisa J. Keay

**Affiliations:** 1 The George Institute for Global Health, Sydney, NSW, AU; 2 Faculty of Medicine, UNSW, Sydney, NSW, AU; 3 Department of Ophthalmology Westmead Hospital, Sydney, NSW, AU; 4 Westmead Institute for Medical Research, Centre for Vision Research, University of Sydney, Sydney, NSW, AU; 5 School of Optometry and Vision Science, Faculty of Science, UNSW Sydney, NSW, AU

**Keywords:** collaborative eye care, health service efficiency, cost saving, task-shifting, chronic eye disease, models of care

## Abstract

**Introduction:**

Glaucoma, a chronic eye disease requires regular monitoring and treatment to prevent vision-loss. In Australia, most public ophthalmology departments are overburdened. Community Eye Care is a ‘collaborative’ care model, involving community-based optometrist assessment and ‘virtual review’ by ophthalmologists to manage low-risk patients. C-EYE-C was implemented at one Australian hospital. This study aims to determine whether C-EYE-C improves access to care and better utilises resources, compared to hospital-based care.

**Methods:**

A clinical and financial audit was conducted to compare access to care and health system costs for hospital care and C-EYE-C. Attendance, wait-time, patient outcomes, and the average cost per encounter were calculated. A weighted kappa assessed agreement between the optometrist and ophthalmologist decisions.

**Results:**

There were 503 low-risk referrals, hospital (n = 182) and C-EYE-C (n = 321). C-EYE-C had higher attendance (81.6% vs 68.7%, p = 0.001); and shorter appointment wait-time (89 vs 386 days, p < 0.001). Following C-EYE-C, 57% of patients avoided hospital; with 39% requiring glaucoma management. C-EYE-C costs were 22% less than hospital care. There was substantial agreement between optometrists and ophthalmologist for diagnosis (k = 0.69, CI 0.61–0.76) and management (k = 0.66, CI 0.57–0.74).

**Discussion:**

C-EYE-C showed higher attendance, and reduced wait-times and health system costs.

**Conclusions:**

Upscale of the C-EYE-C model should be considered to further improve capacity of public eye services in Australia.

## Introduction

Glaucoma is a chronic eye disease that if left untreated can progressively lead to permanent vision loss or blindness [1]. For patients at risk or diagnosed with glaucoma, clinical guidelines recommend routine assessment by an eye-care provider, such as an ophthalmologist, to monitor progression or to initiate timely medical or surgical treatment [1,2].

By 2020, glaucoma is estimated to affect 76 million people worldwide [3]; with associated annual costs to the Australian health system of $AU415 million [4], increasing to $AU784 million by 2025 [5]. At the same time, Australia’s ageing population is expected to see an increase in chronic eye disease [6], further stretching eye-care services and increasing health system costs [4,7].

The Australian health system has a mixed model for providing ophthalmic care, with patients able to access both public and private services. However, patients face barriers to care, with private clinics having high out-of-pocket costs [8] and public hospitals, which are free-of-charge under national health insurance [9,10], often having long appointment wait-times [11]. Ophthalmology services require referral by a primary care provider, such as general practitioner or optometrist, and inappropriate or poorly targeted referrals can cause additional burden to public waitlists. For example, half of the patients referred for routine cataract were discharged at initial assessment because surgery was not needed [12].

Public ophthalmology departments in the United Kingdom (UK) have partnered with community-based optometrists to implement vertical integrated care or ‘collaborative care’ schemes purposed to triage and manage low-risk glaucoma patients [13,14,15,16,17]. These innovative models of care involve an optometrist conducting standardised eye assessment and imaging [13,15,16,17]; a clear referral pathway to the hospital clinic for treatment on indication [15]; and a ‘virtual’ assessment by hospital ophthalmologists to determine clinical need [13,15]. Models such as the Community and Hospital Allied Network Glaucoma Evaluation Scheme (CHANGES) [15] and the Cambridge community Optometry Glaucoma Scheme (COGS) [13] have improved efficiency through a reduction in hospital appointments (8% and 49.5% respectively) which enabled earlier access to specialist care and treatment for those in need. Furthermore, optometrist decisions were found to be in good agreement with ophthalmologist review [13,15,17].

Two European studies have assessed the cost of collaborative models compared to hospital care using a mix of patient, hospital, and health system perspectives. Lower direct and indirect costs for patients were commonly observed in community settings [16,18]. Coast et al (1997) found that the costs per patient visit were lower in community settings [16]. Conversely, Sharma et al (2012) demonstrated higher community costs per visit due to larger overheads and low patient volume [18]. These studies also observed higher annual costs due to the optometrist facility costs and more frequent visits compared to hospital; and lost opportunity costs from private revenue [16,18].

In Australia, there are few collaborative care models where optometrists become formally involved in the assessment and management of glaucoma and other diseases [19,20,21,22]. Like the European studies [13,15,16,17,18], Australian models have shown optometrists to reach good clinical agreement with ophthalmologists [20], hospital appointments can be avoided [19,20,21], and wait-times shortened [19,22]. However, these models are not wide-spread within Australia, and there is little evidence regarding sustainability or costs for the health system.

In 2017, the Westmead Ophthalmology Department (Westmead Eye) introduced a collaborative care model for management of low-risk glaucoma [23] called, Community Eye Care (C-EYE-C). C-EYE-C is a vertical integrated care model that is governed by the hospital and supported by community-based optometrists.

This study aims to determine whether the C-EYE-C model of care for newly referred patients improves access to care and better utilises healthcare resources compared to standard hospital care services.

## Methods

### Study design

A temporal observational evaluation compared the access to care and costs at the first occasion of service for low-risk glaucoma patients under two different models of care i) hospital ophthalmology outpatient clinic and ii) Community Eye Care (C-EYE-C). The first occasion of care included one or more visits to settle on a final diagnosis and treatment plan.

### Setting

Westmead Eye is a public hospital, ophthalmology outpatient department located in western Sydney, Australia. Patients are referred to the hospital by primary care or eye care providers, and regardless of care pathway all referrals are triaged to determine appointment allocation based on clinical priority.

#### Models of care

The two models of care for low-risk glaucoma patients are detailed in **Box 1**.

**Box 1: Two different care pathways for newly referred glaucoma patients (first occasion of care)**

**Table:** 


A) Standard hospital model of care		B) C-EYE-C model of care	

i)	Referral triage (hospital clinician, usually a nurse)	i)	Appointment booking (hospital administration)
ii)	Appointment booking (hospital administration)	ii)	Appointment booking (hospital administration)
iii)	Appointment check-in processing (hospital administration)	iii)	History-taking, clinical examination and imaging* and preliminary diagnosis and management decision recorded (community based optometrist)
iv)	History-taking, screening and imaging* (orthoptists)	iv)	Batch review of patient records to confirm or amend diagnosis and management for all patients assessed at C-EYE-C (hospital ophthalmologist)
v)	Clinical examination (ophthalmologist)	v)	Clerical and file processing (hospital administration)
vi)	Check-out and file processing (hospital administration)	vi)	Patients with clinical need are booked into a hospital glaucoma clinic


* Recommended procedures include contact tonometry, ocular coherence tomography (OCT) and automated perimetry (SITA 24-2 threshold) with a Humphries visual field analyser (VF).

Both models involve referral triage, history-taking, imaging, eye examination, and clerical processing by hospital staff.

Standard hospital care is delivered at the outpatient clinic with examination by an ophthalmologist and support from nurses and orthoptists.

In January 2017, Community Eye Care (C-EYE-C) superseded standard hospital care. C-EYE-C was delivered at a community-based (e.g. shopping centre) optometry practice. An optometrist completed the imaging, examination, and preliminary decision regarding management plan and diagnosis. Assessments were transferred to the hospital using batch store-and-forward for ‘virtual’ review by a consultant ophthalmologist to confirm patient outcomes, or amend as necessary; ensuring all patients receive appropriate management.

### Participants

Participants were identified through a consecutive review of new low-risk glaucoma referrals received by Westmead Eye. For standard hospital care, referrals were included over the period of October 2013–April 2016. C-EYE-C referrals were included from April 2016–October 2017, and implementation of C-EYE-C was delayed until January 2017 due to planning for transition of the service, employment of project staff and consultation with stakeholders. Those not meeting the clinical inclusion criteria (Supplementary Table 1 [24]) or declining an appointment were excluded.

### Data collection

#### Clinical audit

A clinical audit of participants’ medical records was conducted for both models of care. De-identified data were collected for demographics, referral details, attendance, wait-time, diagnosis (low risk glaucoma suspect, high risk glaucoma suspect, early glaucoma, stable early glaucoma, moderate glaucoma, stable moderate glaucoma, advanced glaucoma, acutely raised IOP, other), and recommended management plan (discharge to community, 6-month review C-EYE-C, 12-month review C-EYE-C, review hospital clinic >3 months, urgent review hospital clinic <1 month). Audit data were stored in a REDcap database for standard care [25] and Microsoft Access for C-EYE-C.

#### Costs and Financial audit

A financial audit of clinic operational costs was conducted to calculate the average cost per patient encounter in 2017 Australian dollars for each model of care from a health system perspective.

For the standard hospital care, the cost of an ophthalmology outpatient encounter was obtained from hospital finance records via the NSW Health Activity Based Management Portal (Version 4.5-Build 18.1) [26]. The method for calculating this is described by the Independent Hospital Pricing Authority [27]. Costs include clinical and non-clinical staff salaries and overheads, imaging equipment, medical supplies, and infrastructure. This cost is used for all ophthalmology outpatient encounters with no further breakdowns available at a subspecialty level, e.g. glaucoma, and is therefore reflective of health system costs.

To obtain the average cost per encounter for the C-EYE-C model, staff salary and operational costs for the hospital and optometrist clinics were collected and apportioned for each patient visit. Following discussion with optometry practices it was determined that practice staff could assess 75 patients per week, based on 40 minute appointments needed for visual fields and eye examination and opening times equalling 50 business hours. Hospital staff time required per patient activity was obtained from hospital managers, and salary costs (including overheads) were calculated using mid-point industry award rates. Salary costs for optometrists were obtained using the median hourly income for optometrists in 2016 [28]. Commercial estimates were collected for rent and utility costs at the optometry clinic and apportioned per encounter. The costs for imaging equipment (OCT and HVF) have been reported previously [29], and were depreciated over five-years to match hospital asset management.

## Analyses

### Descriptive

Referral details, demographics, and visit outcomes for diagnosis and recommended management plan were compared between the two models of care.

### Access to care

The attendance rate and wait-time from referral to first appointment was calculated for each model. Hospital wait-list avoidance attributable to the C-EYE-C model was calculated using the number of patients attending C-EYE-C that did not require a hospital follow-up appointment.

### Clinical concordance

For the C-EYE-C model, the proportion of agreement between the optometrist diagnosis and management recommendation and the ophthalmologist virtual review was calculated.

### Cost comparison and sensitivity analysis

The difference in the average cost per patient encounter was compared between models of care. For the C-EYE-C model, patients requiring a follow-up for ophthalmologist intervention (i.e. <3months) at Westmead Eye within the first encounter incurred an additional cost of an abbreviated hospital visit, since imaging had already been completed.

One-way sensitivity analyses were performed on key parameters to determine the impact on the results. Variables tested included the proportion of patients requiring hospital follow-up, variations in all staff costs and repeated for administrative staff (compared to standard hospital care costs), the time per optometrist consultation, and the number of weekly appointments available at community optometrists.

## Statistical analysis

STATA software V15.1 was used for analyses. Chi-Squared or Fisher’s exact tests were used as appropriate to compare categorical data between the models, such as patient attendance or diagnosis. Medians and interquartile range were used to describe wait-times, since the models of care had non-normal distributions. Continuous data were compared using a Mann-Whitney U test. For the C-EYE-C model, an absolute agreement and a weighted kappa statistic with a 95% confidence interval were used to measure interrater agreement between the optometrist and ophthalmologist. Viera & Garret (2005) [30] have defined kappa correlations above 0.61 as ‘substantive’.

## Ethics

Western Sydney Local Health District Human Research Ethics and Scientific Advisory Committee approved this study (5374QA).

## Results

Over the study period (2013–2017) a total of 503 new patients were referred to Westmead Eye with low-risk glaucoma. Referrals mainly came from by optometrists (73.0%) and GPs (20.1%).

There were 182 referrals allocated to standard hospital care, and 321 to C-EYE-C, booked from 1 January 2017.

**Figure1:**
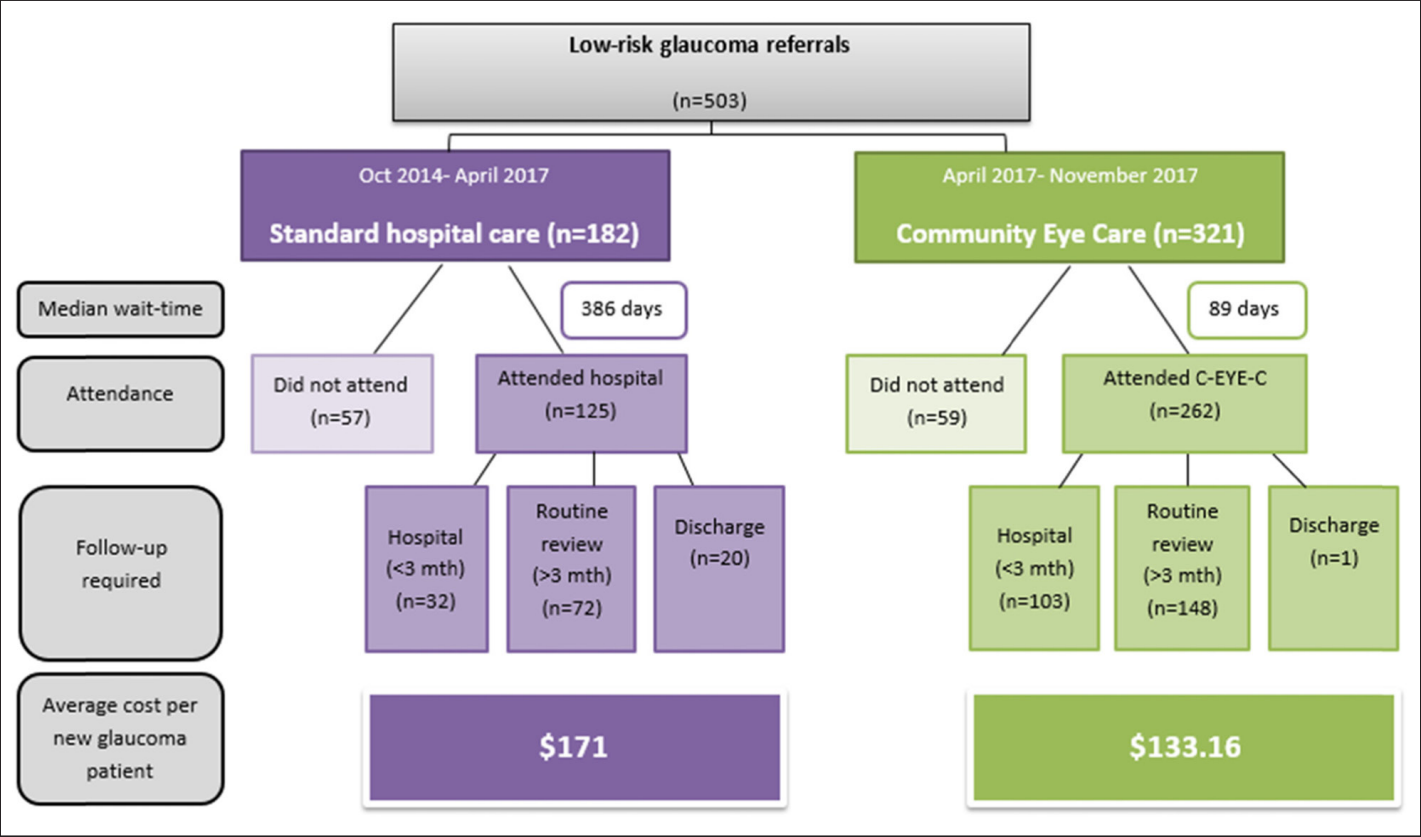
Low-risk glaucoma referrals and patient outcomes under two different models of care 2014–2017.

Referral and patient outcomes for each model of care are displayed in Figure 1.

The mean age of a patient attending an appointment was 63.8 years overall (SD ± 13.97); 64.2 years (SD ± 14.8) for hospital care and 63.6 years (SD ± 13.58) for C-EYE-C.

Overall, equal proportions of females and males attended (50.6% Vs 49.4%), comparable for standard care (50.4% Vs 48.8%) and C-EYE-C (48.5% Vs 51.5%).

Table 1 shows the demographics, access to care, visit outcomes, and costs for each model of care.

**Table 1:** Low-risk glaucoma referrals and patient outcomes under two different models of care 2014–2017.

	Standard Hospital Care	C-Eye-C	P-value

**Low-risk glaucoma referrals received**	**182**	**321**	–
*Referral dates*	October 2013 to April 2016	April 2016 to October 2017	–
*Mean age at referral*	64.2 years (SD ± 14.80)	63.6 years (SD ± 13.58)	p = 0.6959^
*Appointment booked dates*	October 2014 to April 2017	January to November 2017	–
*Appointment attendance (n, %)*	125 (68.7%)	262 (81.6%)	0.001*
*Median wait-time between referral and first appointment (days, IQR)*	386 (IQR: 267–428)	89 (IQR: 53–170)	**<0.001~**
**Glaucoma diagnosis at first appointment**			p < 0.001*
*Glaucoma* + *other ocular pathology*	13 (10.4%)	2 (0.8%)	–
*Glaucoma only*	28 (22.4%)	127 (48.5%)	–
*Glaucoma Suspect*	54 (43.2%)	109 (41.6%)	–
*Not Glaucoma/Other*	22 (17.6%)	18 (6.9%)	–
*Not recorded*	8 (6.4%)	6 (2.3%)	–
**Recommended patient management at first appointment**			P < 0.001*
*Urgent hospital (<1 month)*	1 (0.8%)	8 (3.1%)	–
*Hospital management required (<3 months)*	31 (24.8%)	95 (36.3%)	–
*Routine management (>3 months)*	72 (57.6%)	148 (56.5%)	–
*Hospital review for another ocular condition*	1 (0.8%)	10 (3.8%)	–
*Discharge from service*	20 (16%)	1 (0.4%)	–
**Average cost per patient at first encounter**	$77.00	$133.16	–

p-value: * Fishers Exact used for categorical data. For continuous data, ^ student’s t-test used for parametric data and ~ Mann-Whitney used for non-parametric data.

### Visit outcomes (Diagnosis and recommended management)

The total proportions of patients diagnosed as a glaucoma suspect, with definitive glaucoma, or glaucoma with additional ocular pathology was high across standard care and C-EYE-C (76% Vs 90.9%, Table 1). Standard care had a higher proportion of patients with no recorded diagnosis in the notes compared to C-EYE-C (6.4% Vs 0.8%). Overall a significant difference in the proportion of patients recorded for each diagnosis category between the two models of care was observed (p < 0.001).

Over half of the patients in both standard care and C-EYE-C (57.6% Vs 56.5%) required routine follow-up (>3 months). However, there was a significant variation between the two models of care for patient management (p < 0.001, Table 1).

### Access to care

#### Attendance patterns

Overall, 387 patients (76.9%) attended an appointment following referral, with a significantly higher attendance at C-EYE-C appointments compared to standard care (81.6% Vs 68.7%, p = 0.001). Reasons for non-attendance were not captured.

#### Wait-time

The C-EYE-C model demonstrated a significantly shorter median wait-time from referral to first appointment of 89 days (IQR 53–170) compared to 386 days (IQR 267–428) for standard care (z = –13.667, p < 0.001.)

#### Hospital waitlist avoidance

There were 148 hospital outpatient appointments avoided by patients that attended the C-EYE-C clinic for the first encounter. Assuming that the outpatient clinic has 14 glaucoma appointments available each week for new patients, then 10.6 weeks of appointments were saved by assessing patients off-site at C-EYE-C.

### Clinical concordance for the C-EYE-C model

For diagnosis of glaucoma, the C-EYE-C optometrists and virtual ophthalmology assessment the absolute agreement for glaucoma diagnosis on the 9-point scale was 68% and a 95% weighted agreement (k = 0.69, CI 0.61–0.76), which is considered substantive agreement [30].

For patient management decisions the absolute agreement on the 5-point scale was 79% of assessments, with a substantive weighted agreement of 95% (k = 0.66, CI 0.57–0.74). There was no systematic bias in the management decisions of different providers. For cases where the optometrist’s recommendation was changed, 7.6% required more urgent care, and 13% less. Numbers of patients discharged did not change.

### Costs analysis

The average cost per patient encounter was $171.00 for the hospital model, and $133.16 for C-EYE-C (Table 2). The smaller cost of C-EYE-C was mostly incurred by lower personnel costs through task-shifting from the ophthalmologist to optometrist. Equipment costs were similar, and administration was centralised at the hospital for both models.

**Table 2:** Health systems costs per patient encounter for newly referred low-risk glaucoma patients in the standard hospital care and C-EYE-C models.

Cost item	Hospital care		Community Eye Care (C-EYE-C)		

	Staff time per patient (mins)	Cost per patient encounter	Staff time per patient (mins)	Cost per patient encounter	

				C-EYE-C clinic	Hospital follow-up if required (<3mth)

**STAFFING**	**83**	**$129.00**	**62**	**$53.58**	**$116.00**
***Administration***	13	$21.00	18	$11.11	$21.00
***Nurse***	2	$13.00	2	$1.53	$13.00
***Orthoptist***	43	$10.00	0	$0.00	
***Optometrist***	0	–	40	$31.24	
***Ophthalmologist***	25	$73.00	2	$3.45	$73.00
***On costs* + *exclude***		$12.00		$6.76	$9.00
**EQUIPMENT/**		**$3.00**		**$4.60**	**N/A**
Imaging (OCT, HVF, iCARE)		$3.00		$4.60	N/A
**INFRASTRUCTURE**		**$38.00**		**$13.81**	**$38.00**
Operating room (includes goods and services and salaries)		$6.00		–	$6.00
Pathology		$1.00		–	$1.00
Pharmacy (goods and services and pathology)		$10.00		3.59	$10.00
Prosthesis		$3.00		–	$3.00
Ward supplies (goods and services)		$18.00		–	$18.00
Rent + utilities (optometrist only)		–		$10.22	–
**Cost per patient (by clinic type)**		**$171.00**		**$71.99**	**$154.00**
**AVERAGE COST PER PATIENT**		**$171.00**		**$133.16**	

* On costs + exclude = superannuation, worker’s compensation, long service leave and annual leave.

The results of the sensitivity analyses are presented in Table 3. The costs estimates were most sensitive to increases in the proportion of patients requiring hospital follow-up (<3 months), since this would directly incur more hospital costs.

**Table 3:** Sensitivity analyses of the Community Eye Care model per patient encounter.

Cost variable tested	Range tested	Cost per C-EYE-C patient encounter	Proportional change in cost compared to standard hospital encounter

*Proportion of patients requiring hospital follow-up <3 months*	5–50%	$80.31–149.61	–53.0% to –12.5%
*Optometrist clinic appointments available per week (40 minute appointments)*	±50%	$129.71–143.10	–24.1% to –16.3%
*Optometrist consultation time*	±50%	$114.10–152.21	–33.3% to –11.0%
*Changes to salary (hospital administration)*	±20%	$129.28–137.03	–24.4% to –19.9%
*Changes to salary (all staffing)*	±20%	$113.19–153.11	–33.8% to –10.5%

## Discussion

This study found that using the C-EYE-C model of care for management of newly referred glaucoma patients resulted in a 9.9-month reduction in appointment wait-times, a 22% reduction in health system costs, and was accompanied by a higher patient attendance when compared to standard hospital care.

Other Australian collaborative care models have demonstrated similar improvements in access to care. For example, the Victorian Optometry-Ophthalmology Workforce Collaboration [19] showed a 12 week reduction in wait-time at 6 months after implementation, resulting in a 196 day appointment wait-time [19]. The C-EYE-C model demonstrated that 57% of patients referred for care avoided a hospital appointment, either requiring routine follow-up at C-EYE-C or being discharged to primary care providers. Bourne et al. (2010) [15] similarly found that co-management between optometrists and hospital ophthalmologists led to a 33% reduction in UK hospital outpatient appointments for new low-risk glaucoma and 8% of all patients. Diversion of low-risk patients to the community can free-up hospital resources for patients with higher clinical need, such as surgical intervention for blinding eye diseases including cataracts.

Previous studies have shown moderate agreements between optometrists and ophthalmologists [13,15,17,20,31] for clinical indicators and diagnosis. The authors attribute this to speciality training and continuous feedback-loops to enhance optometrist’s clinical skills. Our agreement is similar to that reported in a UK study by Wright & Diamond (2015) [31] where the weighted agreement between the optometrist and an ophthalmologist in a virtual assessment using similar diagnostic categories for 24,257 cases was 87% (kappa = 0.69.) However, the C-EYE-C model achieved similarly substantial agreement using standardised clinical protocols and continuous feedback alone. Crucially this demonstrates that clinical standards of care were achieved by the C-EYE-C optometrists. Furthermore, this suggests that Australian optometry training is sufficient for involvement in glaucoma collaborative management. Aligning with reported opinions of Australian optometrists involved in a similar model of care [21].

Ophthalmologist oversight in patient management remains a fundamental component for collaborative care to ensure that patients receive appropriate and timely care. Under the C-EYE-C model, 39% of patients required a subsequent hospital appointment within 3 months. Other Australian models involving direct ophthalmologist oversight during the assessment demonstrate a range in patients requiring hospital follow-up may range (from 28% [19] to 11%) [22]. A commonality across C-EYE-C and other models of care, such as the Cambridge Optometry Glaucoma Scheme is streamlined access to hospital services which is enabled through ophthalmologist ‘virtual’ review and centralised hospital governance.

Beyond service efficiency improvements, the C-EYE-C model also demonstrated a 22% cost saving for the Australian health system, which remains even when patient volume increases or decreases. The saving is mainly due to task shifting from an ophthalmologist to an optometrist. Scalability is only impacted when a much higher proportion of patients require specialist intervention during the first encounter. Although this study did not explore the complexities of Australian health care financing, community optometry services are currently funded by items listed in the national health insurance scheme (MBS). The availability of financial incentives for community practitioners and integrated care would be critical for sustainability. Further, the resources saved by C-EYE-C could be redirected toward additional services or treatments. Expansion of C-EYE-C or similar models to cover additional chronic or blinding eye conditions also needs to be investigated, as it is likely that similar efficiencies could be possible. In the Australian health care system there are integrated models of care within other clinical areas, such as antenatal shared care, which have been successfully adopted and scaled to national levels, supported by national pregnancy care guidelines [32] and the introduction of an MBS incentive for participating practitioners in 2005 [33].

Despite the wide spread use of collaborative models across the UK, few studies have assessed cost efficiencies. Coast et al’s (2009) study demonstrated a lower cost per encounter for community-based schemes compared to hospital care. However, the authors also found that annual costs for community schemes were 23% more due to higher practice costs, fewer patients per clinic, and more frequent annual testing. Ophthalmologist oversight in the C-EYE-C model ensures that patients are seen at similar intervals to standard care, thus annual costs would not be impacted by this.

Optometrists have also reported lost opportunity costs from dedicating time to collaborative care compared to revenue they could have received from refractive or spectacles services [16]. However, an increase patient volume at the optometry clinics could negate any losses [16]. In Australia optometrists can attract revenue for glaucoma management through the Medicare Benefit Schedule. To overcome lost opportunity costs optometry practices could increase staffing to enabling continued revenue through refractive and spectacle services without increasing practice overheads. The large costs of equipment may also influence an optometrists’ decision to participate [21]. Thus if additional incentives are needed to encourage optometrist participation, these could be funded through the savings derived from C-EYE-C.

Several limitations are noted. Firstly, it is acknowledged that randomisation of patients across the two models of care would have provided a higher level of evidence than an observational design. Since the evaluation was conducted within a real-world, complex health system a controlled trial was unsuitable; however the same inclusion criteria was used for referrals in each time period, and the generalisability of results are supported by comparable patient outcomes in each model of care. Secondly, the impact of external activities was not measured. For example, during the standard care period multiple service improvement activities were undertaken, including implementation of a referral template and guidelines. Thirdly, the cost analysis used a health system perspective, but did not assess opportunity costs for clinicians, or include patient perspectives, such as out-of-pocket costs. However, previous studies have been able to demonstrate that community-based services can reduce patient out-of-pocket costs [16,18]. Finally, without an evaluation of patient-reported experiences the outcomes observed for these models cannot easily be explained. For example, higher attendance rates at C-EYE-C could be due to shorter wait-times, and patients remembering appointments or not seeking alternate care. Alternatively, several Australian studies have demonstrated a patient preference for community-based services because of reduced travel and high satisfaction with optometrist care [19,21]. It should be noted that estimates for wait-time reductions are conservative, since C-EYE-C did not commence until 2017 and referrals from 2016 accrued a longer wait-time than those received after the clinic opened. Wait-time reduced to as low as 25 days in 2017.

## Conclusion

The C-EYE-C model of care improves access to public ophthalmology services, freeing-up capacity of ophthalmologists to treat patients with higher clinical needs. Importantly the cost-savings shown for C-EYE-C provide a strong economic justification for the health system to scale up this model of care. Despite the success in a large metropolitan hospital, further exploration is a needed to understand how similar models will perform in different settings, for example underserved Aboriginal populations, regional areas with limited coverage of ophthalmologists, or for management of other chronic eye diseases. Furthermore, longitudinal studies are needed to determine how such models influence the burden of disease and macro health economic impacts. With ageing populations and rising chronic disease burden the demand for eye care services will also grow, and integrated models of care such as C-EYE-C are necessary to make healthcare sustainable.

## Additional File

The additional file for this article can be found as follows:

10.5334/ijic.4642.s1 Supplementary Table 1. C-EYE-C clinical inclusion and exclusion criteria for new referrals and follow up patients- glaucoma collaborative management [24].
