# Supplementary material for: Improving Patient Access and Reducing Costs for Glaucoma with Integrated Hospital and Community Care: A Case Study from Australia
Source: Int J Integr Care. 2019 Nov 6;19(4):5. doi: 10.5334/ijic.4642 (PMC6838764; doi:10.5334/ijic.4642)
Supplement: Supplementary Table 1. — C-EYE-C clinical inclusion and exclusion criteria for new referrals and follow up patients- glaucoma collaborative management [24]. [file ijic-19-4-4642-s1.pdf]

*Supplementary Table 1*

**C-EYE-C clinical inclusion and exclusion criteria for new referrals and follow up patients- glaucoma collaborative management [24]**

| | **Glaucoma** |
|---|---|
| *Inclusion* | **Stable glaucoma:**<br>• No change in the management of the patient's glaucoma for 1-2 years<br>• No new progressive visual deterioration, such as a drop in acuity or progression of visual field defect over last 1-2 years<br>• Stable intraocular pressure well controlled on current drug regime (monotherapy, e.g. Xalacom, Duotrav, ganfort)<br>• No progressive optic disc thinning for 1-2 years. This should preferably be based on good quality optic disc photography or OCT<br>• Monitoring for ocular hypertension |
| *Exclusion* | • Newly diagnosed glaucoma<br>• Narrow angles, angle closure glaucoma requiring gonioscopy<br>• Unstable glaucoma<br>    o Chronic or acute angle closure<br>    o Progressive optic nerve changes, expanding nerve fibre layer defect, enlarging cup, new disc haemorrhage, and rim thinning<br>    o Progressive visual field changes, progressive visual field defect, confirmed by repeat testing<br>    o >3mmHg IOP change from last visit (i.e. beyond regression to the mean)<br>    o Clear VF (based on Hodapp Parish Anderson criteria - see Asia Pacific Glaucoma Guidelines Vol 3) or OCT progression on reliable testing (>4 micron change on OCT) or photo disc changes, i.e. new notch or haemorrhage<br>• IOP >30<br>• Complex ocular pathology/ secondary glaucoma (except for PXF or PDS)<br>• Monocular patients<br>• Visual field loss of ≥12dB or within 10 degrees of fixation<br>• Target IOP <12mmHg<br>• Patients requiring surgery for glaucoma, e.g. trabeculectomy or tube implant |
| *Additional exclusion criteria* | • Patients requiring interpretation services (unless available at optometry clinic)<br>• Justice Health patients<br>• Patients ineligible for Medicare, such as non-resident<br>• Patients with known infectious disease e.g. tuberculosis<br>• Patients unable to cooperate e.g. dementia<br>• Hospital in-patients<br>• Patients <16 years of age |
